# Combined forest and soil management after a catastrophic event

**DOI:** 10.1007/s11629-019-5890-0

**Published:** 2020-10-09

**Authors:** Augusto Zanella, Jean-François Ponge, Anna Andreetta, Michael Aubert, Nicolas Bernier, Eleonora Bonifacio, Karine Bonneval, Cristian Bolzonella, Oleg Chertov, Edoardo A. C. Costantini, Maria De Nobili, Silvia Fusaro, Raffaello Giannini, Pascal Junod, Klaus Katzensteiner, Jolantha Kwiatkowsk-Malina, Roberto Menardi, Lingzi Mo, Safwan Mohammad, Annik Schnitzler, Adriano Sofo, Dylan Tatti, Herbert Hager

**Affiliations:** 1 Dipartimento TESAF, Università degli Studi di Padova, Viale dell’Università 16, 35020 Padova, Legnaro (PD), Italy; 2 Museum National d’Histoire Naturelle, CNRS UMR 7179, 4 avenue du Petit Château, 91800 Brunoy, France; 3 Università degli Studi di Firenze Dipartimento di Scienze della Terra (DST) Piazzale delle Cascine, 15 - 50144 Firenze, Italy; 4 URA IRSTEA/EA 1293 — FR CNRS 3730 SCALE, UFR Sciences et Techniques, Université de Rouen, 76821 Mont Saint Aignan cedex, France; 5 Museum National d’Histoire Naturelle, CNRS UMR 7179, 4 avenue du Petit Château, 91800 Brunoy, France; 6 Dipartimento di Scienze Agrarie, Università degli Studi di Torino, Forestali e Alimentari, Largo P. Braccini 2, 10095 Grugliasco (TO), Italy; 7 École supérieure des Arts Décoratifs de Strasbourg, 67082 Strasbourg, France; 8 Dipartimento TESAF, Università degli Studi di Padova, Viale dell’Università 16, 35020 Padova, Legnaro (PD), Italy; 9 Prof. Emeritus, Dr. habil. Ecology, Albert Schweitzer Str. 20, 26129 Oldenburg, Germany; 10 Accademia dei Georgofili, Logge degli Uffizi of Florence, 50122 Florence, Italy; 11 Accademia Nazionale di Agricoltura, Via Castiglione, 11, 40124 Bologna BO, Italy; 12 Department of Agricultural, Food, Environmental and Animal Sciences, University of Udine, via delle Scienze 209, 33100 Udine, Italy; 13 Dipartimento DAFNAE, Università degli Studi di Padova, Viale dell’Università 16, 35020 Padova, Legnaro (PD), Italy; 14 Accademia italiana di scienze forestali, Piazza Tommaso Alva Edison, 11, 50133 Firenze, Italy; 15 Service de la faune, des forêts et de la nature (SFFN) Route des Chéseaux 9, 2017 Boudry, Switzerland; 16 Institute of Forest Ecology, Dept. of Forest and Soil Sciences, University of Natural Resources and Life Sciences (BOKU) Vienna, Peter Jordanstr. 82, 1190 Vienna, Austria; 17 Faculty of Geodesy and Cartography, Department of Spatial Planning and Environmental Sciences, Warsaw University of Technology, Politechniki 1 Sq., 00-661, Warsaw, Poland; 18 Centro Studi Ambiente Alpino, Università degli Studi di Padova, Via F. Ossi, 41, 32046 San Vito di Cadore (BL), Italy; 19 School of Geographical Sciences, Guangzhou University, Guangzhou, 510006 P. R. China; 20 Institute of Land Use, Technology and Regional Development- Faculty of Agricultural and Food Sciences and Environmental Management-University of Debrecen, 4032 Debrecen, Böszörményi út 138, Hungary; 21 Université de Lorraine, Cité Universitaire, 57000 Metz, France; 22 Department of European and Mediterranean Cultures: Architecture, Environment, Cultural Heritage (DiCEM)], Università degli Studi della Basilicata, Via Lanera 20, 75100 Matera, Italy; 23 Haute école des sciences agronomiques, forestières et alimentaires HAFL, Länggasse 85, 3052 Zollikofen, Switzerland; 24 Institute of Forest Ecology, Dept. of Forest and Soil Sciences, University of Natural Resources and Life Sciences (BOKU) Vienna, Peter Jordanstr. 82, 1190 Vienna, Austria

**Keywords:** Vaia storm, Wind damages, Soil organic carbon, Soil functioning, Humus form, Climate change

## Abstract

At the end of October 2018, a storm of unprecedented strength severely damaged the forests of the eastern sector of the Italian Alps. The affected forest area covers 42,500 ha. The president of one of the damaged regions asked for help from the University of Padua. After eight months of discussion, the authors of this article wrote a consensus text. The sometimes asper debate brought to light some crucial aspects: 1) even experienced specialists may have various opinions based on scientific knowledge that lead to conflicting proposals for action. For some of them there is evidence that to restore a destroyed natural environment it is more judicious to do nothing; 2) the soil corresponds to a living structure and every ecosystem’s management should be based on it; 3) faced with a catastrophe, people and politicians find themselves unarmed, also because they rarely have the scientific background to understand natural processes. Yet politicians are the only persons who make the key decisions that drive the economy in play and therefore determine the near future of our planet. This article is an attempt to respond directly to a governor with a degree in animal production science, who formally and prudently asked a university department called “Land, Environment, Agriculture and Forestry” for help before taking decisions; 4) the authors also propose an artistic interpretation of facts (uncontrolled storm) and conclusions (listen to the soil). Briefly, the authors identify the soil as an indispensable source for the renewal of the destroyed forest, give indications on how to prepare a map of the soils of the damaged region, and suggest to anchor on this soil map a series of silvicultural and soil management actions that will promote the soil conservation and the faster recovery of the natural dynamic stability and resilience.

**Electronic Supplementary Material:**

Supplementary material is available for this article at 10.1007/s11629-019-5890-0 and is accessible for authorized users.

## Electronic Supplementary Material

Supplementary material, approximately 155 KB.
