# Supplementary material for: Combined forest and soil management after a catastrophic event
Source: J Mt Sci. 2020 Oct 9;17(10):2459–84. doi: 10.1007/s11629-019-5890-0 (PMC7545024; doi:10.1007/s11629-019-5890-0)
Supplement: Supplementary file 1 — Supplementary material, approximately 155 KB. [file 11629_2019_5890_MOESM1_ESM.pdf]

The following Appendixe (Appendixes 1) is the Electronic Supplementary Material of the article entitled "Combined forest and soil management after a catastrophic event" at https://doi.org/10.1007/s11629-019-5890-0

Appendix 1 European soil regions and subregion (EDGI 2016), altitude, soil references (IUSS Working Group WRB 2015; Soil Survey Staff 2014) and humus systems (Zanella et al. 2018e) examples. From Agenzia Regionale per la Prevenzione e Protezione Ambientale del Veneto (2019), synthetic overview.

Soil region	Soil subregion	Soil subregion description	Altitude (m)	Examples of common soils	Soil diagnostic horizons	Humus system (diagnostic horizons)
37.1	MA1	Soils formed from moderately competent silicate lithotypes. They are located on high slopes and at the top of the main mountain ranges, at medium energy of the relief, with common coverings of glacial and slope deposits.	1900-2500	Moderately deep, stony soils, with moderate profile differentiation and locally with moderate translocation of aluminium and iron sesquioxides in depth (Dystric Cambisols - Dystrudepts) and moderately deep, stony soils, with high profile differentiation, with sesquioxides and organic substance translocation in depth (Entic Podzols - Humicryods)	A(AE)-Bs-CO; A-Bhs-Bs-BC-C	Moder (OL, zoOF, zoOH or szoOH, sgA) or Mor (OL, nozOF or szoOH, msA or sgA or absence of A horizon)
	MB1	Soils formed from moderately competent silicate lithotypes. They are located on medium and low slopes of main mountain ranges and secondary chains, at medium energy of the relief, with extensive coverage of glacial and slope deposits	1000-1900	Moderately deep, stony soils, with moderate profile differentiation and moderate translocation of aluminium and iron sesquioxides in depth (Dystric Cambisols - Dystrudepts)	AE-Bs-BC-C	Moder (OL, zoOF, zoOH, miA)

(-To be continued-)

Appendix 1 European soil regions and subregion (EDGI 2016), altitude, soil references (IUSS Working Group WRB 2015; Soil Survey Staff 2014) and humus systems (Zanella et al. 2018e) examples. From Agenzia Regionale per la Prevenzione e Protezione Ambientale del Veneto(2019), synthetic overview.
(-Continued-)

Soil region	Soil subregion	Soil subregion description	Altitude (m)	Examples of common soils	Soil diagnostic horizons	Humus system (diagnostic horizons)
34.3	DA1	Soils formed from very competent carbonate lithotypes. They are located on high slopes and at the top of the main mountain ranges, high-energy relief, with common coverings of glacial and slope deposits.	1700-2800	Thin, very stony soils with low profile differentiation and accumulation of organic matter on surface (Rendzic Leptosols - Cryrendolls)	OA-AC-C	Tangel (OL, zoOF, zoOH or szoOH, meA or absence of A)
	DA3	Soils formed from moderately competent carbonate lithotypes. They are located on high slopes and at the top of the main mountain ranges, at medium energy of the relief, with common coverings of glacial and slope deposits.	1700-2500	Thin, very stony soils, with low profile differentiation, on steep and / or eroded surfaces (Rendzic Leptosols - Cryrendolls) and moderately deep, stony soils, with high profile differentiation, with leaching of clays on stable morphologies (Skeletic Luvisols - Inceptic Hapludalfs).	OA-AC-C; A-E-Bt-BC-C	Tangel (OL, zoOF, zoOH or szoOH, meA or absence of A) or Amphi (OL, zoOF, zoOH, meA or maA)
		Soils formed from moderately competent carbonate lithotypes. They are located on high slopes and at the top of the main mountain ranges, at medium energy of the relief, with common coverings of glacial and slope deposits.	1700-2500	Thin, very stony soils, with low profile differentiation, on steep and / or eroded surfaces (Rendzic Leptosols - Cryrendolls) and moderately deep, stony soils, with high profile differentiation, with leaching of clays on stable morphologies (Skeletic Luvisols - Inceptic Hapludalfs).	OA-AC-C; A-E-Bt-BC-C	Tangel (OL, zoOF, zoOH or szoOH, meA or absence of A) or Amphi (OL, zoOF, zoOH, meA or maA)

(-To be continued-)

Appendix 1 European soil regions and subregion (EDGI 2016), altitude, soil references (IUSS Working Group WRB 2015; Soil Survey Staff 2014) and humus systems (Zanella et al. 2018e) examples. From Agenzia Regionale per la Prevenzione e Protezione Ambientale del Veneto(2019), synthetic overview.
(-Continued-)

Soil region	Soil subregion	Soil subregion description	Altitude (m)	Examples of common soils	Soil diagnostic horizons	Humus system (diagnostic horizons)
34.3	DB1-2	Soils formed from very competent carbonate lithotypes. They are located on medium and low slopes of main mountain ranges and secondary chains, high-energy relief, with extensive coverage of glacial and slope deposits.	500-2100	Thin, very stony soils with low profile differentiation (Rendzic Phaeozems - Typic Haprendolls).	A-(AC)-C	Mull (OL, zoOF, maA) or Amphi (OL, zoOF, zoOH, meA or maA)
	DB3	Soils formed from moderately competent silicate lithotypes. They are located on medium and low slopes of main mountain ranges and secondary chains, at medium energy of the relief, with extensive coverage of glacial and slope deposits.	1000-2000	Moderately deep, stony soils, with moderate profile differentiation and moderate translocation of aluminium and iron sesquioxides in depth (Dystric Cambisols- Spodic Dystrudepts).	AE-Bs-BC-C	Mull (OL, zoOF, maA) or Moder (OL, zoOF, zoOH, miA)
	DB4	Soils formed from moderately competent carbonate lithotypes. They are located on steep surfaces and / or subject to erosive phenomena, of medium and low slopes of main mountain ranges and secondary chains, at medium energy of the relief, with extensive coverage of glacial and slope deposits	400-2300	Deep, stony soils with high profile differentiation, with clay accumulation in depth (Skeleti-Cutanic Luvisols - Inceptic Hapludalfs) and moderately deep, stony soils with moderate profile differentiation (Haplic Cambisols -Typic Udorthents)	A-E-Bt-BC-C; (OA)-A-Bw-BC-C	Mull (OL, zoOF, maA) or Amphi (OL, zoOF, zoOH, meA or maA)

(-To be continued-)

Appendix 1 European soil regions and subregion (EDGI 2016), altitude, soil references (IUSS Working Group WRB 2015; Soil Survey Staff 2014) and humus systems (Zanella et al. 2018e) examples. From Agenzia Regionale per la Prevenzione e Protezione Ambientale del Veneto(2019), synthetic overview.

(-Continued-)

Soil region	Soil subregion	Soil subregion description	Altitude (m)	Examples of common soils	Soil diagnostic horizons	Humus system (diagnostic horizons)
34.3	DB5-6	Soils formed from competent carbonate lithotypes. They are located on stable surfaces of medium and low slopes of main mountain ranges and secondary chains, at medium energy of the relief and with extensive coverage of glacial and slope deposits	400-2000	Soils from moderately deep to deep, stony, with high profile differentiation, with accumulation of clay in depth (Cutanic Luvisols - Typic Hapludalfs), or with moderate profile differentiation with evident hydromorphy (Endogleyic Cambisols - Aquic Eutrudepts)	A-(BE)-Bt-C; A-Bw-(BCg)-Cg	Mull (OL, zoOF, maA)
	SA1-2-3-4	Soils on surfaces from sub-floors to undulating and slopes, in hard limestone, locally affected by karst phenomena	600-1800	Moderately deep soils, on rock, with high profile differentiation, with deep clay accumulation (Leptic Luvisols - Inceptic Hapludalfs) on wooded surfaces and thin soils, on rock, with moderate profile differentiation, with accumulation of organic substance on the surface (Leptic Cambisols - Typic Eutrudept); Soils on weakly concave surfaces affected by colluvial and alluvial troughs. Deep, stony soils with high profile differentiation and clay accumulation in depth (Luvic Phaeozems - Typic Argiudolls)	A-Bt-R; A-Bw-BC-R; A-(EB)-Bt	Mull (OL, zoOF, maA) or Amphi (OL, zoOF, zoOH, meA or maA)
	SD1	Soils on high-slope slopes formed by hard limestone with abundant debris deposits on the foot and in the watersheds.	300-1400	Thin soils, on rock, with low profile differentiation, with accumulation of organic substance on the surface (Epileptic Phaeozems - Lithic Hapludolls)	OA-A-R	Amphi (OL, zoOF, zoOH, meA or maA)

(-To be continued-)

Appendix 1 European soil regions and subregion (EDGI 2016), altitude, soil references (IUSS Working Group WRB 2015; Soil Survey Staff 2014) and humus systems (Zanella et al. 2018e) examples. From Agenzia Regionale per la Prevenzione e Protezione Ambientale del Veneto(2019), synthetic overview.
(-Continued-)

Soil region	Soil subregion	Soil subregion description	Altitude (m)	Examples of common soils	Soil diagnostic horizons	Humus system (diagnostic horizons)
34.3	SD2	Soils on slopes and on narrow ridges developed on marly limestone with medium-high slopes and dense drainage network.	300 -1700	Thin soils, on rock, with low profile differentiation, with accumulation of organic substance on the surface, partial decarbonation (Endoleptic Phaeozems - Entic Hapludolls) on very steep eroded slopes and moderately deep soils, on rock, with high profile differentiation, with clay accumulation in depth (Cutanic Luvisols - Typic Hapludalfs), on stable morphologies	A-AC(AB)-(Bw)/R; A-Bt-BC-C	Amphi (OL, zoOF, zoOH, meA or maA) or Mull (OL, zoOF, maA)
	SI1	Soils on valley incisions in dolomite with predominantly steep slopes.	300-2000	Thin soils, on rock, with moderate profile differentiation, with accumulation of organic substance on the surface (Haplic Cambisols - Inceptic Haprendolls)	A-Bw-(BC)-C	Mull (OL, zoOF, maA)
	SI2	Soils on valley incisions in limestones, with steep slopes	200 -2000	Very thin soils, on rock, with low profile differentiation, with accumulation of organic substance on the surface (Rendzic Phaeozems - Entic Hapludolls) on steep slopes, and moderately deep soils, very stony, with moderate profile differentiation, with accumulation of organic substance on the surface (Haplic Cambisols - Inceptic Haprendolls) on scree slopes	A-AB(Bw)-R; A-Bw-(BC)-C	Amphi (OL, zoOF, zoOH, meA or maA) or Mull (OL, zoOF, maA)
34.3	SI3	Soils on valley incisions, escarpments, small basins in marly limestone (Biancone) and subordinately to marls with regular rounded slopes with strong slope.	300-1300	Thin soils, on rock, with low profile differentiation, with accumulation of organic substance on the surface, partial decarbonation (Epileptic Phaeozems - Lithic Hapludolls) on very steep eroded slopes and moderately deep soils, on rock, with high profile differentiation, with clay accumulation in depth (Cutanic Alisols - Ultic Hapludalfs) on stable morphologies	A-R; A-EB-Bt	Amphi (OL, zoOF, zoOH, meA or maA) or Mull (OL, zoOF, maA)

Appendix 2 Countries of production, quantity and price of softwood imported to China in 2018 (Zhu 2019)

Countries	Softwood quantity (million m^3)	Softwood price ($/$m^3$)
New Zealand	17.29	141
Russian	7.95	117
The United States	5.03	166
Australia	4.13	126
Canada	2.53	184
Japan	0.92	134
Uruguay	2.09	124
Others	1.64	-
Total	41.6	139

References

Agenzia Regionale per la Prevenzione e Protezione Ambientale del Veneto (2019) Legenda della Carta dei Suoli del Veneto in Scala 1: 250,000. Versione (2019). Regione Veneto. En: Regional Agency for Environmental Prevention and Protection of Veneto. Soil Map Legend 2019. https://www.arpa.veneto.it/temi-ambientali/suolo/conoscenza-dei-suoli/carta-1-250.000/leg_250k.pdf/view

The legend briefly describes all the cartographic units. These are inserted in a hierarchical structure that includes four levels, in accordance with what is proposed at the national level for the "Map of the Soils of Italy on a scale of 1: 250,000". The first level is that of soil regions (L1 -soil regions), represented in the map in a box on a scale of 1: 5,000,000; these are the result of the national re-elaboration of the map of the soil regions of Europe, prepared by the European Soil Bureau and attached to the "Manual of Procedures for a Georeferenced Database of European Soils". The second level, represented in a box on a scale of 1: 1,000,000, corresponds to the provinces of soils (L2 - soil subregions). The third level, soil systems (L3 - great soilscapes), is identified by different colors in the legend of the 1: 250,000 scale map. The fourth level, which corresponds to that of the cartographic units (L4 - soil subsystems - soilscapes), is shown on the map only as an abbreviation within the individual delineations as the high number does not allow unambiguous identification through different colors.

The classification system used is the World Reference Base for Soil Resources, 2006 version, and the 2010 USDA Soil Taxonomy.

EDGI (2016) EuroGeoSurvey. European Geoscience for Society [WWW Document]. Geol. Surv. Organ. Eur. URL http://www.europe-geology.eu/soil/soil-map/soil-regions/ (accessed on 2020-09-11)

IUSS Working Group WRB (2015) World Reference Base for Soil Resources 2014, update 2015 International soil classification system for naming soils and creating legends for soil maps., World Soil Resources Reports No. 106. Food and Agriculture Organization of the United Nations, Rome, Italy. https://doi.org/10.1017/S0014479706394902

Soil Survey Staff (2014) Keys to Soil Taxonomy by Soil Survey Staff, 12th edition., 12th ed, Soil Conservation Service. United States Department of Agriculture, Natural Resources Conservation Service, Washington, DC.

Zanella A, Ponge JF, Jabiol B, et al. (2018) Humusica 1, article 5: Terrestrial humus systems and forms - Keys of classification of humus systems and forms. Applied Soil Ecology 122: 75-86. https://doi.org/10.1016/j.apsoil.2017.06.012

Zhu GQ (2019) Changes and problems in China's import and export market of wood and wood products in 2018. International Wood Industry (1): 18-22. (In Chinese). https://doi.org/10.3969/j.issn.1671-4911.2019.01.006
